# Effects of soil nitrogen on diploid advantage in fireweed, *Chamerion angustifolium* (Onagraceae)

**DOI:** 10.1002/ece3.4797

**Published:** 2018-12-26

**Authors:** Alex L. Bales, Erika I. Hersch‐Green

**Affiliations:** ^1^ Microbiology Department University of Massachusetts Amherst Massachusetts; ^2^ Department of Biological Sciences Michigan Technological University Houghton Michigan

**Keywords:** adaptation, autopolyploidy, fitness, global change, resource allocation strategies, soil nitrogen

## Abstract

In many ecosystems, plant growth and reproduction are nitrogen limited. Current and predicted increases of global reactive nitrogen could alter the ecological and evolutionary trajectories of plant populations. Nitrogen is a major component of nucleic acids and cell structures, and it has been predicted that organisms with larger genomes should require more nitrogen for growth and reproduction and be more negatively affected by nitrogen scarcities than organisms with smaller genomes. In a greenhouse experiment, we tested this hypothesis by examining whether the amount of soil nitrogen supplied differentially influenced the performance (fitness, growth, and resource allocation strategies) of diploid and autotetraploid fireweed (*Chamerion angustifolium*). We found that soil nitrogen levels differentially impacted cytotype performance, and in general, diploids were favored under low nitrogen conditions, but this diploid advantage disappeared under nitrogen enrichment. Specifically, when nitrogen was scarce, diploids produced more seeds and allocated more biomass toward seed production relative to investment in plant biomass or total plant nitrogen than did tetraploids. As nitrogen supplied increased, such discrepancies between cytotypes disappeared. We also found that cytotype resource allocation strategies were differentially dependent on soil nitrogen, and that whereas diploids adopted resource allocation strategies that favored current season reproduction when nitrogen was limiting and future reproduction when nitrogen was more plentiful, tetraploids adopted resource allocation strategies that favored current season reproduction under nitrogen enrichment. Together these results suggest nitrogen enrichment could differentially affect cytotype performance, which could have implications for cytotypes’ ecological and evolutionary dynamics under a globally changing climate.

## 1 INTRODUCTION

Whole genome duplication (polyploidy) commonly occurs in plants and is an important evolutionary process contributing to both intraspecific genetic and phenotypic variation (Levin, 2002; Otto & Whitton, 2000) and to the formation of new species (Masterson, 1994). In plants, polyploidy has been shown to be correlated with increased cell, flower, and seed sizes, reduced growth rates, and qualitative and quantitative changes to primary and secondary metabolites (Dhawan & Lavania, 1996; Evans, 1967; Hull‐Sanders, Johnson, Owen, & Meyer, 2009; Kennedy, Sabara, Haydon, & Husband, 2006; Maherali, Alison, & Husband, 2009; Otto & Whitton, 2000), all of which can influence interactions between plants and their abiotic and biotic environments. For example, relative to related diploids, polyploids can differentially attract pollinators (Segraves & Anneberg, 2016; Segraves & Thompson, 1999; Thompson, Nuismer, & Merg, 2004), exhibit different patterns of resistance and tolerance to insect and pathogen damage (Guegan & Morand, 1996; Nuismer & Otto, 2004; Nuismer & Thompson, 2001; Segraves & Anneberg, 2016; Thompson et al., 2004), and are more or less tolerant of temperature (Liu et al., 2011), light (Fukuda, 1967), and water stressors (Guo et al., 2016; Liu et al., 2011; Maherali et al., 2009). Polyploidy may also contribute to invasiveness and adaptations to novel habitats (te Beest et al., 2012). Because abiotic and biotic environmental conditions are changing at unprecedented rates at both regional and global scales (Jamieson, Trowbridge, Raffa, & Lindroth, 2012; Shaver et al., 1992; Vitousek et al., 1997), examination of whether such environmental changes disproportionately favor some cytotypes over others could increase our understanding of the ecological and evolutionary consequences of genome duplication in a globally changing environment.

Human activities have resulted in elevated levels of reactive (bioavailable) nitrogen in terrestrial and aquatic ecosystems worldwide (Vitousek et al., 1997; Vitousek, Hättenschwiler, Olander, & Allison, 2002). Furthermore, it is predicted that nitrogen pools will continue to increase over the next century due to contributions from both rising atmospheric nitrogen deposition rates (e.g., reactive nitrogen in wet and dry forms is expected to increase by a factor of 2.5 over land by 2,100; Lamarque et al., 2005) and rising temperatures, which have been shown to facilitate nitrogen mineralization rates especially in cold boreal and arctic regions (Rustad et al., 2001; Shaver et al., 1992). While nitrogen enrichment is often associated with increases in plant growth and ecosystem primary productivity, it is also a major driver of declining plant species diversity, causing radical changes in multitrophic level biodiversity patterns and ecosystem properties (Clark & Tilman, 2008; Hautier, Niklaus, & Hector, 2009; Stevens, Dise, Mountford, & Gowing, 2004; Stevens et al., 2010, 2015). Declines in plant species diversity patterns from nitrogen enrichment are thought to result, in part, from changes in dominance hierarchies, which in turn relies on differences in nitrogen requirements and the efficacy of nitrogen use by different individuals and species. A plethora of research has shown that primary producers vary in both their requirements for and responses to available nitrogen (De Schrijver et al., 2011; Wooliver et al., 2014; Xia & Wang, 2008) and that nitrogen enrichment can disproportionately favor some species over others (Chapin, 1980; Suding et al., 2005; Tamm, 1991; Tilman, 1982, 1986). However, it is not known whether nitrogen enrichment influences the performance of some plants over others based upon their genome size or number of chromosomes (ploidy level).

Here, we evaluate the overall hypothesis that polyploidy, or a difference in the number of chromosomes among closely related plants, influences individual performance responses under different soil nitrogen supplies. Nitrogen is an integral component of chloroplasts, histone packaging proteins, cell membranes, and nucleic acids (Sterner & Elser, 2002) and therefore plays a role in both growth and reproduction. Because polyploids have more chromosomes per cell and larger cells and genomes than closely related diploids, it has been predicted that polyploids and organisms with larger genomes should require more nitrogen and should be more restricted by nitrogen scarcities than diploids or organisms with smaller genomes (Cavalier‐Smith, 2005; Guignard et al., 2016; Leitch & Bennett, 2004; Lewis, 1985). Although there has yet to be a direct test of whether nitrogen scarcities differentially influence the performance of diploid over closely related polyploid plants, there is evidence showing that nitrogen availability can both selectively influence genome structure and the ecological sorting of plant communities. For example, ecological nitrogen limitation and increased photosynthetic nitrogen use requirements have both been shown to exert selection on the base pair composition of plant DNA and RNA in favor of less nitrogen‐costly substitutions (Acquisti, Elser, & Kumar, 2009; Kelly, 2018) and have been proposed as a driver of genome size reduction (Kang, Wang, & Huang, 2015). Furthermore, field studies in grasslands have found that plants with larger genomes and polyploids make up a greater proportion of the aboveground plant biomass in plots fertilized with both nitrogen and phosphorus as compared to aboveground biomass in plots given one nutrient or no supplemental nutrients (Guignard et al., 2016; Šmarda et al., 2013).

Lifetime fitness is difficult to accurately and completely measure (Primack & Kang, 1989; Younginger, Sirová, Cruzan, & Ballhorn, 2017), as it depends upon the combined influence of many traits, and organisms can adopt different strategies to maximize fitness under different conditions (Chapin, Schulze, & Mooney, 1990; Harper & Ogden, 1970). For instance, plant species grown or adapted to nutrient‐poor habitats have been shown to allocate more biomass into belowground structures at the expense of aboveground structures (Dennis & Johnson, 1970) and/or to invest a higher percentage of their aboveground growth into reproductive tissues relative to vegetative tissues (Tilman & Cowan, 1989). In perennial organisms, investment into aboveground growth and reproductive tissues can favor competition for light and current season fitness, whereas higher investment into belowground biomass and resource storage can favor overwintering and future season fitness gains (Chapin et al., 1990). Polyploidy has been shown to alter the resource allocation patterns of plants responding to changes in water availability (Guo et al., 2016), yet it is not known whether polyploidy might also influence resource allocation strategies and long‐term plant fitness in response to changes in soil nitrogen availabilities.

**Figure 1:**
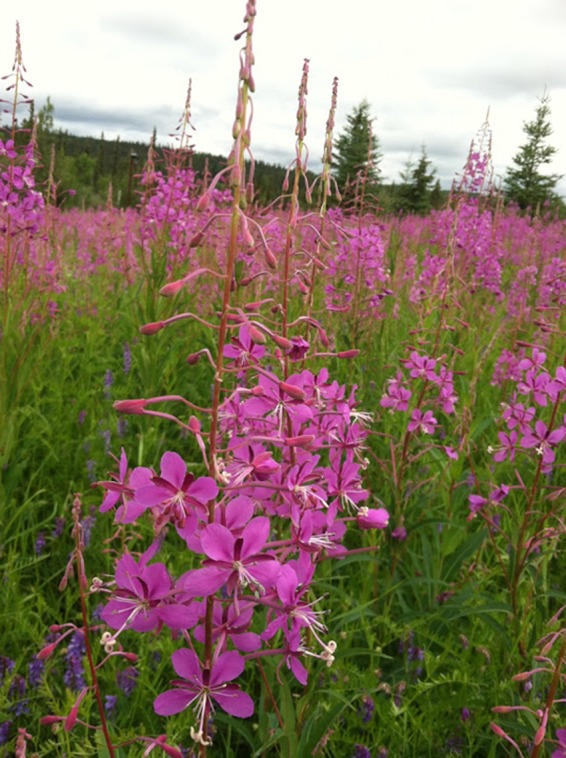
Study species, *Chamerion angustifolium* (Onagraceae)

To begin to assess whether diploids and closely related polyploids (e.g., alternative cytotypes) differ in their fitness and growth responses to soil nitrogen concentrations, we conducted a series of greenhouse experiments using field‐collected seeds of *Chamerion angustifolium* (fireweed—Figure 1). In *C. angustifolium,* polyploidy occurs from genome duplication within a species’ lineage (autopolyploidy) as opposed to genome duplication occurring concurrently with hybridization (allopolyploidy). We believe that by studying an autopolyploid, we are able to more precisely isolate potential responses due to increased genome size rather than to increased heterozygosity arising from the merger of two evolutionary distinct genomes that is associated with allopolyploidy. We grew diploid and autotetraploid fireweed under three different levels of soil nitrogen (low, medium, high) and measured performance responses and resource allocation strategies. Because we expected that diploids should require less nitrogen to support their smaller genomes and cell sizes, we predicted that they would have higher fitness measures (in terms of flower, pollen, and seed production) than tetraploids when nitrogen is scarce but that this “diploid advantage” might be reduced or reversed when soil nitrogen is more plentiful and no longer a limiting factor. Alternatively, we may find that polyploids are not limited under nutrient scarce conditions as they may not require more nitrogen for synthesis and growth if, for example, there are tradeoffs in cell size and cell number per area of tissue (Cavalier‐Smith, 2005) or if polyploids are more efficient at using nitrogen. We also predicted that because fireweeds are perennial plants, that they would invest more into current season reproductive efforts (more aboveground biomass and reproductive measures) when nitrogen was more plentiful and more into storage (more belowground biomass and nutrient accumulation) when nitrogen was less available. In general, we found support for our main hypothesis in that diploids performed better than autotetraploids under low nitrogen conditions but that such “diploid advantage” disappeared under more nitrogen‐rich conditions. We also found that when soil nitrogen was lower plants, especially autotetraploids, invested more into aboveground biomass than they did when soil nitrogen was higher. We discuss the ecological and evolutionary implications of our findings with respect to fireweed distribution patterns and to predicted increases in soil nitrogen availability.

## 2 MATERIALS AND METHODS

### 2.1 Plant material

Fireweed (*Chamerion angustifolium *(L.) Holub*.* ssp. *angustifolium*, Onagraceae) is an herbaceous perennial plant species that is widely distributed throughout much of the northern hemisphere. Fireweed exists as diploid (2*n* = 2*x* = 36 chromosomes), autotetraploid (2*n* = 4*x* = 72 chromosomes), and occasionally triploid (2*n* = 3*x* = 54 chromosomes) cytotypes and is a model system for studying the ecological and evolutionary consequences of autopolyploidy (Husband & Schemske, 1998; Kennedy et al., 2006; Maherali et al., 2009; Schemske, 2000; Thompson, Husband, & Maherali, 2014). Historical census data from North America reported that diploid fireweed was mostly restricted to far northern latitudes (above 60°N latitude, including most of Alaska), tetraploids mostly occurred in more southern latitude regions, and mixed cytotype populations were common in between and along the Rocky Mountain range (Mosquin & Small, 1971). However, we surveyed over 1,000 fireweed plants throughout interior Alaska and found that in contrast to historical censuses, tetraploids dominated and mixed cytotype populations were frequent in far northern latitude sites (Supporting Information Appendix S1, Table S1). High‐latitude regions are experiencing especially elevated rates of soil nitrogen mineralization due to the influence of climate warming on microbial activity (Rustad et al., 2001; Shaver et al., 1992), and such changes can alter species distribution patterns (Bret‐Harte, Shaver, & Chapin, 2002; Sturm, Racine, & Tape, 2001; Zamin, Bret‐Harte, & Grogan, 2014). Therefore, we examined whether elevated bioavailable nitrogen might differentially influence fireweed cytotype performance and potentially contribute to future shifts in fireweed cytotype distribution patterns.

Prior to experimentation, we generated diploid and tetraploid full‐sibling maternal seed families (genetic lines) by growing fireweed from seeds collected in 2013 from two mixed cytotype sites in interior Alaska (Supporting Information Appendix S1, Table S1) under identical nutrient, soil, temperature, and water conditions in a greenhouse at Michigan Technological University (Department of Biological Sciences, Houghton, MI). To do this, we hand‐pollinated plants such that all plants received pollen from a different plant of the same ploidy level (outcross hand‐pollinated). Ploidy level of all parental plants prior to hand pollinations was determined by estimating plant nuclear 2C DNA content using flow cytometry following a modified protocol of Baldwin and Husband (2013); fireweed plant nuclear 2C DNA content is highly correlated with chromosome number (Husband & Schemske, 1998). Briefly, approximately 1 cm^2^ of silica dried fireweed leaf tissue was co‐chopped with approximately 1‐cm^2^ fresh leaf issue of *Solanum lycospersicum* (an internal standard, 2C DNA content = 1.96; Doležel, Greilhuber, & Suda, 2007) in a modified DeLaat's nuclei isolation buffer with propidium iodide stain (Supporting Information Appendix S1). Flow cytometry data were collected with an Accuri C6 flow cytometer and analyzed using CFlow Plus Analysis software (Accuri Inc., Ann Arbor, MI) to determine the cytotype of each parental plant (diploid, triploid, or tetraploid; all triploids were excluded from experimentation).

### 2.2 Experimental design

We germinated 18 seeds from seven diploid and seven tetraploid genetic lines (experimentally created as described above) in a growth chamber at 24°C in 2,500‐cm^3^ pots containing a 1:1 mixture of vermiculite to Sunshine soil mix #4 (Sun Grow Horticulture Ltd., Vancouver, BC, Canada). After three weeks, we moved plants to the greenhouse, grew them under a 16:8‐hr light:dark cycle, and assigned each plant to one of three nitrogen (N) treatments (low, medium, or high; six replicates per seven genetic lines for each of two cytotypes and three nitrogen treatments = 252 total plants). Nitrogen was supplied to pots in three doses, and each dose provided 0.67, 6.7, or 67 mg of ammonium nitrate per pot, which based on a dry soil weight equated to final nitrogen concentrations of approximately 12, 120, and 1,200 ppm of N per pot (µg N g^−1^ soil). We applied nitrogen treatment so as to minimize leaching, although actual soil N concentrations may have been lower due to plant use. The low and medium soil N concentrations were chosen to reflect measurements of reactive soil nitrogen (NH_4_‐N + NO_3_‐N) reported near our seed collection sites in interior AK (~10–220 ppm µg N g^−1^ soil, Clein & Schimel, 1995; Gordon, Tallas, & Cleve, 1987; Kielland, McFarland, Ruess, & Olson, 2007; Van Cleve, Barney, & Schlentner, 1981; Van Cleve, Oechel, & Hom, 1990), whereas the high N treatment was selected to resemble N‐rich conditions that could be indicative of patchy areas of excessive N deposition (e.g., from animal excretion or agricultural fertilizer runoff). All plants also received equal amounts of phosphorous (P; 15 ppm supplied as potassium monophosphate), potassium (K; 250 ppm supplied as potassium sulfate), and micronutrients (0.615‐ml Fertilome chelated liquid iron and other micronutrients; Voluntary Purchasing Groups, Inc., Bonham, TX). Plants received a mixed nutrient solution of N, P, and K three times during the third, fourth, and fifth weeks of growth and a single application of the micronutrients during the sixth week of growth; all plants received these treatments as a 20 ml solution, which was readily absorbed by the soil to minimize potential nutrient leaching. Additions of P, K, micronutrients, and moisture throughout the experiment were also administered in such a fashion as to alleviate any potential plant growth limitations by these other resources and to minimize leaching or runoff. Plants were randomly arranged in the greenhouse and rotated weekly to minimize potential nonrandom environmental effects imposed by variable greenhouse conditions. Because spontaneous whole genome duplications can occur, we also verified the ploidy level of all plants used in the experiment with flow cytometry as described above and data from nine triploids (N = 2, 4, and 3 from low, medium, and high nitrogen treatments, respectively) were excluded from statistical analyses.

### 2.3 Fitness, flowering phenology, and biomass estimates

To assess whether soil N supplied differentially favors one cytotype over another, we measured traits associated with plant fitness and growth. We evaluated plant fitness by measuring total flower production and the average number of pollen grains per anther as proxies for males fitness (Lehtilä & Strauss, 1999; Sutherland & Delph, 1984) and seed production from selfed and outcrossed pollinations as proxies for female fitness (Strauss, Conner, & Lehtila, 2001; Strauss, Conner, & Rush, 1996). Flower production was measured by counting the total number of flowers produced during a plant's life cycle. The average number of pollen grains per anther was estimated by collecting one anther near dehiscence from two flowers on each plant, allowing anthers to dehisce, suspending them in 500 µl of 95% ethanol, and averaging the number of pollen grains counted in two 10‐µl aliquots (Kearns & Inouye, 1993). Total seed production was estimated by conducting hand pollinations (performed by rubbing two anthers worth of dehiscent pollen evenly across receptive stigmas) to mimic self‐ and outcross pollinations normally facilitated by bees and other insects in natural populations (Kennedy et al., 2006; Schemske, 2000). For hand pollinations, we selected four flowers on each plant; two flowers received pollen from a different flower on the same plant (selfed treatment) and two flowers received pollen from different plants within the same ploidy and soil N treatment (outcrossed treatment). Mature fruits were collected, and seeds were counted with a Pfeuffer Contador II seed counter (Pfeuffer GmbH, Kitzingen, Bavaria, Germany) to obtain an average number of seeds produced per fruit for both selfed and outcrossed treatments. In order to estimate a plants’ potential maximum seed production (MSP), we multiplied the mean number of seeds produced per fruit from outcrossed pollinations by the total number of flowers per plant (seeds per fruit × number of flowers). We did not use seed production from self‐pollinations to calculate MSP because we found that both cytotypes produced significantly more seeds in the outcross treatments and we wanted to measure maximum potential under different nitrogen supplies. Because flowering phenology can affect total flower production and also be influenced by soil N availability and ploidy (Husband & Schemske, 1998; Zhang, Niu, Liu, Jia, & Du, 2014), we counted the number of days from seed germination to the first open flower produced on each plant (flowering phenology).

We measured aboveground and belowground biomass as direct measures of plant growth and as indirect measures of competitive abilities; aboveground biomass can reflect competitive ability for aboveground resources, as larger plants are more competitive for light (Tilman, 1988), whereas belowground biomass can reflect overwintering potential and competitive ability for soil water and nutrients (Bloom, Chapin, & Mooney, 1985; Chapin et al., 1990). To measure biomass, we harvested mature plants, separated each into above‐ and belowground portions, dried all material at 60°C, and weighed each portion of all plants to the nearest mg. Plants that showed no signs of flowering (~59%) were harvested at approximately 16 weeks of growth, and plants that did flower were harvested immediately following seed collection at around 18 weeks of growth.

### 2.4 Resource allocation tradeoffs

To examine whether cytotypes differ in their resource allocation strategies and/or whether soil nitrogen levels influence these differences, we calculated four ratios that describe patterns of resource allocation: (a) above‐ to belowground biomass, (b) total aboveground N to total belowground N, (c) maximum seed production to total plant biomass, and (d) maximum seed production to total plant N. We estimated above‐ to belowground biomass (Shoot‐mass: Root‐mass) by dividing root dry weight by shoot dry weight. Because diploid and tetraploid fireweed could differ in the relative total amount of N stored in their above‐ versus belowground tissues, we also measured the percentage of N in each tissue in order to estimate the total amount of N in shoots (shoot‐N = shoot dry weight × percent N in shoots) and in roots (root‐N = root dry weight × percent N in roots). This allowed us to determine whether cytotypes differ in their proportion of total N invested in above‐ versus belowground tissues (Shoot‐N:Root‐N). To measure the percentage of N in each tissue, we separately homogenized entire dried shoot and root biomass portions to a fine powder with a ball mill and used an elemental analyzer (ECS 4010, Costech Analytical Technologies Inc., Valencia, CA) to determine the percentage of N in a 5 mg subsample of each tissue. Lastly, we calculated ratios of maximum seed production (MSP) to total plant biomass (MSP: Total‐mass) and to total plant N (MSP: Total‐N), to compare the relative efficiency of diploid and tetraploid fireweed at generating reproductive output per unit of total plant biomass and total plant N, respectively. We calculated total plant biomass and total plant N as the sum of shoot‐mass and root‐mass and sum of shoot‐N and root‐N, respectively, and divided maximum seed production by each of these sums.

### 2.5 Statistical analysis

We examined whether ploidy level (diploid, tetraploid), soil N treatment (low, medium, high), and genetic line (nested within ploidy level) influenced plant performance metrics using a series of statistical tests. In all analyses, all factors were treated as fixed effects, although repeated analyses treating genetic line (nested within ploidy level) as a random effect did not yield significantly different results. In logistic regression analyses, we examined the effect of genetic line for each ploidy level by manually accounting for the deviance due to genetic line, and in all other analyses, we examined the effect and significance of ploidy level by examining the variance among ploidy levels relative to the between‐genetic line variance. Logistic regression analyses were conducted in R version 3.5.1 (R Core Team, 2014), and all other analyses were conducted in JMP version 13.0 (SAS Institute Inc., Cary, NC). In all analyses, model assumptions of normality and homogeneity of variances were tested by statistically examining model residuals and transformations were made when necessary, Tukey's HSD tests were used to compare for significant differences among means when nitrogen treatment (three levels) was significant (*p* < 0.05), and independent contrasts between ploidy levels within each N treatment level were conducted when an interaction between ploidy and N treatment was significant (*p* < 0.05).

First, because many plants (~59%) did not flower, we used logistic regression to examine whether ploidy level, soil N treatment, their interaction, and/or genetic line (nested within ploidy level) influenced the likelihood of flowering. Next, given that a plant flowered, we used ANOVA models to examine whether soil N treatment, ploidy level, their interaction, and/or genetic line influenced seven traits associated with plant fitness and growth: total flower production, pollen production, the number of seeds produced per fruit from self and outcross pollinations, potential maximum seed production, and/or total, above‐ and belowground biomasses.

To assess whether flowering phenology might impact total flower production and/or maximum seed production (MSP), we tested for correlations between days to first flower and these variables. Next, we used an ANOVA model with ploidy level, soil N treatment, their interaction, and genetic line to determine whether cytotypes produce flowers at separate times and whether any differences in flowering phenologies could be influenced by soil nitrogen concentrations. Lastly, to assess whether cytotypes potentially differ in their resource allocation strategies under different nitrogen environments, we tested whether ploidy level, soil N treatment, their interaction, and/or genetic line influenced fireweed's Shoot‐mass: Root‐mass (log‐transformed), Shoot‐N:Root‐N (log‐transformed), MSP: Total‐mass, and MSP: Total‐N.

## 3 RESULTS

### 3.1 Ploidy and soil nitrogen influence plant flower, seed, and fruit production

**Figure 2:**
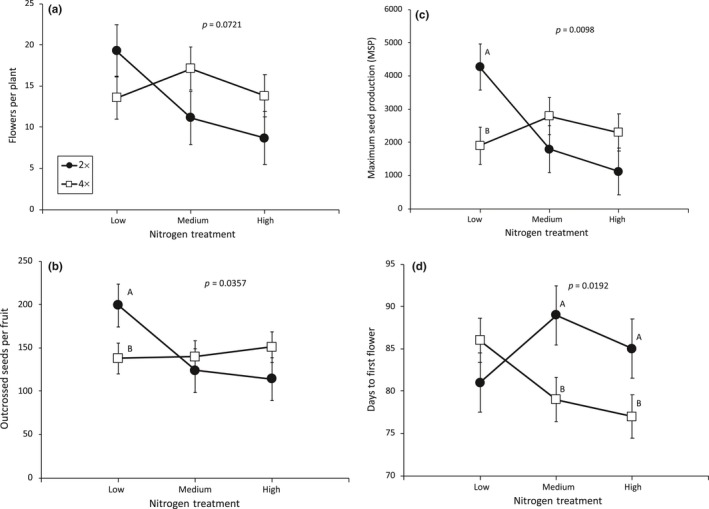
Ploidy and soil nitrogen treatment interactions on (a) the number of flowers produced per plant, (b) the number of outcrossed seeds produced per fruit, (c) maximum seed production (MSP), and (d) flowering phenology of fireweed (*Chamerion angustifolium*). We report *p*‐values from ploidy × soil N treatment interactions, LS means ±1 *SE*'s, and significant independent contrasts between cytotypes within nitrogen treatments when significant (*p* < 0.05) with separate letters; full statistical details are reported in Table 2

On average, cytotypes significantly differed in their likelihood of flowering with 51% of tetraploids flowering as compared to only 31% of diploids flowering, although the likelihood of flowering did also vary between genetic lines (Table 1). Neither soil N treatment nor an interaction between ploidy and soil N treatment significantly affected the likelihood of flowering (Table 1). For plants that did flower, no tested factors significantly affected flower production (Table 2a; Figure 2a) or pollen production (Table 2b).

**Table 1:** Results from logistic regression showing the effects of ploidy, soil N treatment, their interaction (P × N), and genetic line (nested within ploidy) on the likelihood of fireweed (*Chamerion angustifolium*) flowering (overall model: *df* = 17, χ^2^ = 70.68, *p* < 0.0001, *R* ^2^ = 0.22); all factors were treated as fixed effects and bold values indicate a significant effect at *α* = 0.05. To examine the effect of genetic line for each cytotype, we manually accounting for the deviance due to each genetic line

Source	*df*	*χ* ^2^	*p*
Ploidy	1	6.87	**0.0088**
Soil N	2	2.18	0.3361
P × N	2	0.86	0.6493
Genetic line (diploid)	6	58.51	**0.0258**
Genetic line (tetraploid)	6	44.18	**<0.0001**

**Table 2:** Results of ANOVAs for the effects of ploidy level (diploid, tetraploid), soil nitrogen treatment (low, medium, high), their interaction (P x N), and genetic line (nested within ploidy) on components of fireweed (*Chamerion angustifolium*) performance, phenology, and growth. All factors were treated as fixed effects, and we report the *F*‐ratio and level of significance (Prob > *F*) for the effect of ploidy relative to the between‐genetic line variance (MS_genetic line_). Overall Model *R* ^2^ values are given for each factor, and bold values indicate a significant effect at *α* = 0.05

Factor	Source	*df*	MS	*F*‐ratio	Prob > *F*
a. Flowers per plant (*R* ^2^ = 0.19)	Ploidy	1	39.96	0.37	0.5543
	Soil N	2	154.55	1.55	0.2193
	P × N	2	271.51	2.72	0.0721
	Genetic line	12	108.65	1.09	0.3822
	Model error	82	99.98		
b. Pollen grains per stamen (*R* ^2^ = 0.35)	Ploidy	1	3,806,822	2.91	0.1138
	Soil N	2	275,320	0.35	0.7053
	P × N	2	1,160,073	1.48	0.2366
	Genetic line	12	1,308,609	1.67	0.0999
	Model error	54	783,459		
c. Selfed seeds per fruit (*R* ^2^ = 0.47)	Ploidy	1	42,390.59	7.19	**0.0200**
	Soil N	2	3,256.16	1.25	0.2923
	P × N	2	990.03	0.38	0.6848
	Genetic line	12	5,895.82	2.27	**0.0173**
	Model error	69	2,600.66		
d. Outcrossed seeds per fruit (*R* ^2^ = 0.21)	Ploidy	1	63.33	0.02	0.8900
	Soil N	2	9,330.37	2.07	0.1336
	P × N	2	15,744.10	3.50	**0.0357**
	Genetic line	12	2,847.97	0.63	0.8071
	Model error	69	4,500.76		
e. Max seed production (MSP) (*R* ^2^ = 0.24)	Ploidy	1	47,844	0.01	0.9118
	Soil N	2	8,674,761	2.02	0.1407
	P × N	2	21,292,278	4.95	**0.0098**
	Genetic line	12	3,709,957	0.86	0.5871
	Model error	69	4,298,506		
f. Days to flower (*R* ^2^ = 0.45)	Ploidy	1	205.48	0.57	0.4648
	Soil N	2	63.02	0.65	0.5256
	P × N	2	403.91	4.16	**0.0192**
	Genetic line	12	360.82	3.71	**0.0002**
	Model error	81	97.21		
g. Total biomass (*R* ^2^ =0.19)	Ploidy	1	3.91	1.26	0.2836
	Soil N	2	0.06	0.06	0.9388
	P × N	2	2.52	2.86	0.0595
	Genetic line	12	3.11	3.53	**<0.0001**
	Model error	223	0.88		
h. Shoot biomass (*R* ^2^ = 0.29)	Ploidy	1	6.16	4.11	0.0654
	Soil N	2	0.84	0.84	0.4329
	P × N	2	1.26	1.26	0.2866
	Genetic line	12	1.50	5.39	**<0.0001**
	Model error	223	0.28		
i. Root biomass (*R* ^2^ = 0.13)	Ploidy	1	0.25	0.43	0.5243
	Soil N	2	0.46	1.51	0.2222
	P × N	2	1.00	3.28	**0.0394**
	Genetic line	12	0.58	1.89	**0.0370**
	Model error	223	0.31		

When receiving self‐pollen, we found that 83% of tetraploids produced fruit as compared to only 61 percent of the diploids (although these differences were not significant). Furthermore, given that a plant produced fruit from self‐pollination, tetraploids, on average, produced significantly more seeds per fruit than diploids (LS mean ± 1*SE* for diploids: 25.1 ± 15.2, tetraploids: 87.2 ± 12.9), and this was despite the observation that genetic lines significantly differed in the average number of seeds produced per fruit from self‐pollinations (Table 2c). Neither nitrogen treatment nor an interaction between nitrogen treatment and plant ploidy significantly affected seed production from selfing (Table 2c).

In contrast, on average across nitrogen treatments in outcross pollinations, both cytotypes were equally likely to produce fruits (83% of diploid and 87% of tetraploid outcrossed flowers produced fruits, respectively), and produced equivalent average numbers of seeds per fruit (Table 2d), and had equivalent maximum seed potential (Table 2e). However, in the different nitrogen treatments, cytotypes did differ significantly in average seed production per fruit and in maximum seed production from outcrossed pollinations (i.e., significant ploidy × soil N interaction; Table 2d,e). Independent contrasts among ploidy levels at the different nitrogen treatments showed that, on average, diploids tended to produce more outcrossed seeds per fruit and have a greater potential to produce seeds than tetraploids when soil nitrogen was low, but that there were no significant differences among cytotypes when soil nitrogen was moderate or high (Table 2d,e; Figure 2b,c).

Soil nitrogen had different effects on cytotype flowering phenology (Table 2f), and in general, diploids flowered significantly later than tetraploids when soil N was moderate to high, but differences in flowering time among cytotypes did not significantly differ when soil N was low (Table 2f, Figure 2d). Observed differences in flowering phenologies between cytotypes and plants in the different soil N treatments likely compounded observed differences in estimated maximum seed production (MSP) between cytotypes in the different N treatments. For example, in general, plants that flowered later produced fewer flowers (*R* ^2^_98_ = 0.30, *p* < 0.001) and seeds (*R* ^2^_91_ = 0.19, *p* < 0.001) than plants that flowered earlier, and the manner in which soil N influenced flowering phenology and thus potential seed production differed between cytotypes. More specifically, when soil N was low, diploids tended to produce flowers earlier than tetraploids (although not significantly, Figure 2d), and this phenology difference likely contributed to estimates of higher seed production (MSP) of diploids under low nitrogen conditions (Figure 2c). In contrast, when soil N was more abundant, tetraploids produced flowers significantly earlier than diploids (Figure 2d), and this likely contributed to estimates of higher seed production (MSP) for tetraploids under the medium and high nitrogen treatments (Figure 2c), thereby negating differences among cytotype in MSP under nutrient enrichment.

### 3.2 Ploidy and soil nitrogen influence plant biomass and resource allocation strategies

Above‐ and belowground biomass and resource allocation strategies can impact plant competitive abilities and ultimately lifetime success. In general, we found that cytotypes did not differ in terms of their total biomass, nor did total biomass increase with increasing soil N (Table 2g). Cytotypes also did not differ in terms of their aboveground biomass (Table 2h), although we found that tetraploids tended to be, on average, slightly larger than diploids in terms of aboveground biomass (LS mean ±1 *SE* for diploids: 1.46 ± 0.05, tetraploids: 1.38 ± 0.05). However, such differences were likely due primarily to differences among certain genetic lines. For example, when the effect of ploidy was tested relative to the residual variance, ploidy level had a significant effect on shoot biomass (*F*_1,82_ = 22.13; *p* < 0.0001), however when the effect of ploidy was tested relative to the between‐genetic line variance, it did not (Table 2h). On average, cytotypes also did not differ in terms of belowground biomass production, although soil N treatment had different effects on belowground biomass production between cytotypes (Table 2i). Specifically, in the high N treatment, diploid root belowground biomass was significantly greater than tetraploids, but under moderate to low soil N, cytotypes did not significantly differ in terms of belowground biomass production (Supporting Information Figure S1). Genetic lines also varied in terms of total, aboveground and belowground biomass production, but no other factors significantly affected biomass production (Table 2g,h,i).

**Figure 3:**
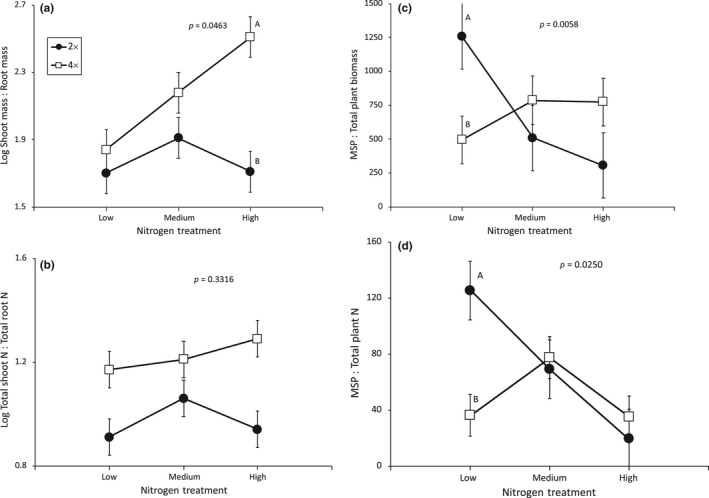
Ploidy and soil nitrogen treatment interactions on fireweed's (*Chamerion angustifolium*) (a) shoot biomass to root biomass (log‐transformed), (b) shoot‐N to total root‐N (log‐transformed), (c) maximum seed production (MSP) to total plant biomass, and (d) maximum seed production (MSP) to total plant N. We report *p*‐values from ploidy × soil N treatment interactions, LS means ±1 *SE*'s, and significant independent contrasts between cytotypes within nitrogen treatments when significant (*p* < 0.05) with separate letters; full statistical details are reported in Table 3

Both ploidy level and N treatment had a significant effect on a plant's relative investment into shoots versus roots, although the effects were dependent upon each other and varied among genetic lines (Table 3a). In general, tetraploids invested a greater proportion of their total biomass into shoots relative to roots (Shoot‐mass: Root‐mass) as compared to diploids (LS mean ± 1 *SE* for diploids: 1.77 ± 0.07, tetraploids: 2.18 ± 0.07), but such differences were only significant under the high soil N treatment (Figure 3a). Tetraploids also invested a greater proportion of total plant N into their shoots relative to roots (Shoot‐N:Root‐N)in comparison with diploids (LS mean ± 1 *SE* for diploids: 2.78 ± 0.12, tetraploids: 3.61 ± 0.17) despite the fact that genetic lines differed in investment patterns, but such differences did not depend upon soil N treatment (Table 3b; Figure 3b). While this result would be expected given the generally larger shoot biomass to root biomass of tetraploids (Table 3a), it is surprising because both tetraploid shoot and root tissues have less N per mg tissue (%N) than diploid shoot and root tissues (Supporting Information Figure S2). No other factors significantly influenced the allocation of biomass or N to shoots versus roots (Table 3a,b).

**Table 3:** Results from ANOVAs testing the effects of ploidy (diploid, tetraploid), soil N treatment (low, medium, high), their interaction (P × N), and genetic line (nested within ploidy) on four resource allocation strategies of fireweed (*Chamerion angustifolium)*: Shoot‐mass:Root‐mass (log‐transformed), Shoot‐N:Root‐N (log‐transformed), maximum seed production (MSP):Total‐mass, and maximum seed production (MSP):Total‐N*. *All factors were treated as fixed effects, and we report the *F*‐ratio and level of significance (Prob > *F*) for the effect of ploidy relative to the between‐genetic line variance (MS_genetic line_). Overall Model *R* ^2^ values are given for each factor, and bold values indicate a significant effect at *α* = 0.05

Factor	Source	*df*	MS	*F*	*p*
a. Log Shoot‐mass:Root‐mass (*R* ^2^ = 0.22)	Ploidy	1	2.45	9.8	**0.0087**
	Soil N	2	0.48	4.52	**0.0180**
	P × N	2	0.37	2.66	**0.0463**
	Genetic line	12	0.25	2.24	**0.0182**
	Model error	223	0.12		
b. Log Shoot‐N:Root‐N (*R* ^2^ = 0.28)	Ploidy	1	2.06	10.30	**0.0075**
	Soil N	2	0.11	1.01	0.3665
	P × N	2	0.12	1.80	0.3316
	Genetic line	12	0.20	1.51	**0.0503**
	Model error	114	0.11		
c. MSP:Total‐mass (*R* ^2^ = 0.25)	Ploidy	1	274.40	>0.00	0.9793
	Soil N	2	567,823	1.38	0.2578
	P × N	2	2,230,880	5.55	**0.0058**
	Genetic line	12	407,788	0.99	0.4647
	Model error	69	410,675		
d. MSP:Total‐N (*R* ^2^ = 0.47)	Ploidy	1	3,749.88	0.18	0.6789
	Soil N	2	5,344.51	2.62	0.0881
	P × N	2	8,450.12	4.15	**0.0250**
	Genetic line	12	21,296.89	0.95	0.5081
	Model error	32	2,037.33		

Cytotypes differed in their allocation to seed production relative to total mass (MSP: Total‐mass), but such differences were dependent upon soil nitrogen treatments (Table 3c). For instance, at the low soil N treatment, diploids had significantly higher maximum seed production potential per mg of plant biomass than tetraploids (independent contrast: *F*_1,69_ = 6.45, *p* = 0.0133), whereas when soil N was medium to high, diploids had lower, although not significantly lower maximum seed production potentials per mg of plant biomass than tetraploids (independent contrast for medium N: *F*_1,69_ = 1.05, *p* = 0. 3,086, high N: *F*_1,69_ = 1.81, *p* = 0.1825; Figure 3c). A similar pattern emerged for investment into maximum seed production relative to total plant N (Table 3d), wherein at the low soil N treatment, diploids had a significantly higher maximum seed production potential per mg of plant N compared to tetraploids (independent contrast: *F*_1,32_ = 9.89, *p* = 0.0036), whereas in the medium and high soil nitrogen treatments cytotypes did not significantly differ (independent contrast for medium N: *F*_1,32_ = 0.09, *p* = 0. 7,661, high N: *F*_1,32_ = 0.211, *p* = 0.6490; Figure 3d).

## 4 DISCUSSION

Deposition of bioavailable nitrogen into ecosystems worldwide has increased dramatically over the past century and is expected to continue to increase well into the future. Global warming, especially in high‐latitude cold regions, is predicted to further increase reactive N in these regions because increasing temperatures are expected to positively affect nitrogen mineralization rates (Bret‐Harte et al., 2002; Chapin, 1991; Nadelhoffer, Giblin, Shaver, & Laundre, 1991; Shaver, Iii, & Gartner, 1986; Shaver & Kummerow, 1992). It is unclear, however, how such increases in soil nitrogen availability will affect plant performance dynamics, species distributions, and the ecological and evolutionary trajectories of plant species. Because nitrogen is a central component of nucleic acids, cell structures, and photosynthetic processes, it was hypothesized that diploids (with smaller genomes) should be favored over polyploids (with larger genomes) when nitrogen is scarce because organisms with smaller genomes are expected to have smaller nitrogen requirements (Cavalier‐Smith, 2005; Guignard et al., 2016; Leitch & Bennett, 2004; Lewis, 1985). Here, we tested this hypothesis using diploid and autotetraploid cytotypes of an herbaceous perennial plant, fireweed, which is common throughout northern temperate regions where nitrogen availability is typically low. Our results suggest that low levels of soil nitrogen may favor diploids over tetraploids and that increases in ecosystem available nitrogen may erase such diploid advantages, perhaps shifting competitive dynamics and ultimately impacting plant population landscape patterns.

### 4.1 Increasing soil nitrogen does not always lead to greater plant productivity

We expected that increasing soil nitrogen would have positive impacts on the productivity of both cytotypes in terms of higher flower and seed production and greater investment of resources into biomass. However, we found that increasing soil nitrogen did not unequivocally lead to greater flower and seed production and to greater investments into biomass. In particular, diploids were actually least productive in terms of flower and seed production under conditions of high soil nitrogen, while tetraploids exhibited less variability for these traits with changing nitrogen conditions (Figures 2a2c). It is not known why diploid flower and seed production were depressed in the high soil treatments, but one explanation is that the excess nitrogen was toxic, as the concentration of N supplied in our high treatments was significantly greater than the levels found in natural populations. While this explanation is plausible, we think that it is unlikely to fully explain our observations here because diploid seed production responses to nitrogen did not significantly differ between the high and medium treatment conditions and the medium level of N was within the range reported near our seed collection sites in interior AK (~10–220 ppm µg N g^−1^ soil, Van Cleve et al., 1981; Gordon et al., 1987; Van Cleve et al., 1990; Clein & Schimel, 1995; Kielland et al., 2007). Furthermore, neither diploid above‐ or belowground biomass was significantly lower when grown in the medium and high nitrogen treatments (Supporting Information Figure S1), which would be expected under toxicity. However, shifts in diploid fireweed's flowering phenology under higher nitrogen conditions likely contributed to their reduced flower and maximum seed potential (MSP) in the medium and high soil nitrogen treatments. For instance, diploids in the low soil nitrogen treatment flowered earlier than diploids in the medium and high soil nitrogen treatments (Figure 2d), and plants that began flowering earlier produced more flowers leading to higher maximum seed production estimates. The effects of soil nitrogen appeared to influence tetraploids’ flowering phenology differently, leading to differences in flower and maximum potential seed production among cytotypes. Specifically, tetraploids in the low soil nitrogen treatment flowered later than those in the medium and high soil nitrogen treatments (Figure 2d) and this phenological shift may have contributed to lower flower production under low soil nitrogen conditions for the tetraploids, further compounding the slightly lower estimates of MSP for tetraploids in the low nitrogen conditions (Figure 2c).

We also found that increased soil nitrogen did not unequivocally lead to significant increases in plant biomass traits (Table 2g,h,i), which was surprising because increased soil nitrogen is most typically associated with greater plant growth and productivity (Chapin, 1991; Shaver et al., 1986; Shaver & Kummerow, 1992; Zhang et al., 2014). However, not all plant species are equally limited by nitrogen (in terms of fitness and growth), and some species can have a competitive advantage under low nitrogen conditions that can disappear with fertilization (Chapin, 1980; Suding et al., 2005; Tamm, 1991; Tilman, 1982, 1986). One explanation for our results is that plants might be differentially limited by the availability of other resources, such as phosphorous, water, or other nutrients, which could influence seed production and biomass accumulation. For instance, phosphorus is also a major component of RNA and associated gene expression, DNA, and other cellular components. Recent field studies have found that polyploid species and species with larger genomes responded more positively to phosphorous fertilization and both phosphorous and nitrogen fertilization relative to diploids and species with smaller genomes (Guignard et al., 2016; Šmarda et al., 2013). If phosphorous was limiting or co‐limiting in our study, this could have partially muted or altered the effects of nitrogen treatments. Although a recent greenhouse study suggested that fireweed cytotypes are not differentially affected by phosphorus supply (Walcyzk, 2018), future studies that consider the co‐limitation of combined resources would increase our understanding of whether cytotypes might differ in their responses to changing nutrient and resource availabilities.

### 4.2 “Diploid advantage” at low but not high soil nitrogen conditions

We hypothesize that tetraploids would be at a disadvantage relative to diploids under low soil nitrogen conditions because tetraploids are expected to require more nitrogen to support their larger genomes, cells, and plant forms and that such disadvantages may disappear under higher nitrogen conditions. However, it was also possible that there could be tradeoffs in cell size and number such that polyploids might not require more nutrients for building blocks at the whole plant level. In congruence with our initial predictions, we found that tetraploids but not diploids were limited, in terms of flower, fruit, and seed production, by nitrogen scarcities. For instance, when soil nitrogen was low, diploids produced more flowers and significantly more seeds than tetraploids, but when soil nitrogen was moderate to high, discrepancies between the cytotypes disappeared (Figure 2b,c). Furthermore, when diploids were grown in low nitrogen soils they also invested significantly more into reproductive output (MSP) relative to vegetative biomass and nutrient storage than tetraploids and such differences among cytotypes also disappeared under more nitrogen‐rich conditions (Figure 3c,d). In areas where soil nitrogen limits plant productivity, plants may have a current season competitive advantage if they are able to produce more seeds and flowers with fewer resources invested into vegetative growth (i.e., high MSP: Total‐mass or high MSP: Total plant N). Our data suggest that diploids might have a competitive advantage over tetraploids in nitrogen‐poor soils, but as bioavailable nitrogen increases, differences among cytotypes might disappear.

One caveat is that our interpretation of differential effects of soil nitrogen on current season fitness depends upon patterns of outcrossing in natural populations. For example, our estimates of maximum seed production (MSP) only considered data from outcrossed pollinations, but differences in selfing rates and seed production from selfing in natural populations would also affect current season fitness. Here, we found that a large proportion of flowers that were self‐pollinated did not produce fruit and/or produced on average less seeds per fruit than flowers that received outcross pollen and that fruit and that seed production discrepancies between self and outcross pollinations were more pronounced for diploids than for tetraploids. However, for neither cytotype did nitrogen conditions affect fruit or seed production from self‐pollinations and thus if patterns of selfing are equivalent for both cytotypes in natural populations than our interpretation that diploids have a competitive advantage over tetraploids in nitrogen‐poor soils is supported. Interestingly, others have reported higher levels of inbreeding depression in diploids relative to tetraploids and/or a breakdown of self‐incompatibility following polyploidization in natural fireweed populations (Husband & Sabara, 2004; Husband & Schemske, 2000).

### 4.3 Implications for a perennial life history and cytotype distributions

Perennial plant species, like fireweed, reproduce over multiple seasons, and thus, their lifetime fitness is maximized by strategies that balance current season growth and productivity with that of future seasons. For instance, when nutrients are limiting, plants may achieve higher lifetime fitness gains by investing more resources into storage for later reproduction when nutrient conditions may be more plentiful and amenable to reproduction. Alternatively, when nutrients are plentiful, perennial plants may experience higher lifetime fitness payoffs by investing more into current reproductive outputs than into storage (Bloom et al., 1985; Chapin et al., 1990; Tilman, 1988). Furthermore, increasing soil nitrogen concentrations has been associated with increased shoot: root ratios (Austin & Austin, 1980; Austin, Groves, Fresco, & Kaye, 1985; Tilman, 1986). Although previous studies have reported that tetraploid fireweed (Husband & Schemske, 1998) and other polyploid species (Eigsti, 1957; Evans, 1967; Liu et al., 2011; Maceira, Jacquard, & Lumaret, 1993) tend to be larger than related diploids, we did not find that tetraploids were significantly larger than diploids (Table 2g,h,i). However, we did find evidence suggesting that cytotypes have different resource allocation strategies that are differentially dependent on soil nitrogen availabilities. For instance, we found that especially under high nitrogen supplies diploids invested a larger proportion of their total biomass and nitrogen into roots relative to shoots than tetraploids (Figure 3a,b, Supporting Information Figure S1). More total nitrogen in roots may provide diploids with an advantage in terms of subsequent years’ productivity and/or an ability to re‐allocate resources if late season reproduction is threatened. Furthermore, when nitrogen is more limiting, diploids invested more into current season reproductive output relative to biomass or total plant nitrogen investment (Figure 3c,d). Together these data suggest that when nitrogen is limiting diploids might invest more into current season growth, whereas when nitrogen is more plentiful, they might be investing more into future season fitness potential. In contrast, tetraploids do not appear to invest as much into storage and future reproduction and instead use additional nitrogen for current season growth and reproduction. In addition, while tetraploids’ shoot‐mass: root‐mass increased with greater soil nitrogen, diploids did not respond to soil nitrogen treatments with the same level of plasticity (Figure 3a). Common garden experiments by Guo et al. (2016) compared the shoot: root of diploid and hexaploid fireweed under three levels of soil water availability and found no detectable change in shoot: root for diploids across water treatments, but significant increases in shoot: root for hexaploids with increasing water availability. These results combined with our results might suggest that polyploids exhibit greater plasticity in responding to changes in resource availabilities as compared to diploids.

Lastly, observed changes in flowering phenology between cytotypes among the different nitrogen treatments could also have consequences for natural populations. We found that diploids flowered earlier than tetraploids when nitrogen was low but flowered later than tetraploids when nitrogen was at higher concentrations (Figure 2d). Previous greenhouse and field studies have also reported that diploid fireweeds begin flowering earlier than co‐occurring tetraploids (Husband & Schemske, 1997, 2000). In mixed populations when cytotypes interbreed, they produce triploid hybrids that are either inviable or that show reduced fitness relative to either diploids or tetraploids; this flowering asynchrony is thought to be one of several reproductive barriers that allow cytotypes to coexist and maintain reproductive isolation from each other (Husband & Schemske, 2000). Here, we found that nitrogen enrichment appeared to completely reverse previously observed patterns in which diploids flowered earlier than tetraploids (Figure 2d). Therefore, increases in bioavailable nitrogen might maintain the reproductive barrier of temporal differences in flowering, although if soil nitrogen concentrations are patchy, then cytotypes may flower in synchrony in some areas leading to hybridization events and a breakdown of reproductive isolation.

## 5 SUMMARY

Our greenhouse experiment allowed us to examine the role of soil nitrogen in regulating the relative performance of diploid and tetraploid fireweed while controlling for extraneous variables commonly occurring in field conditions that could interact to influence the results of fertilization experiments. Our results suggest that increases in soil nitrogen from global changes may have significant influences on the performances, resource allocation strategies, flowering phenologies, and tissue nutrient profiles of cytotypes, potentially impacting the adaptive value of genome duplication in natural populations. While the high nitrogen treatment may be unrealistic in terms of reflecting likely future nitrogen conditions in natural populations, we chose it to determine whether excess nitrogen would have similar consequences for the plants as they do from more moderate nitrogen levels (such as the medium N treatments). It is worth noting that for most responses measured, neither cytotype differed in terms of their performance and growth responses between the medium and high nitrogen treatments (Figures 2 and 3). Nevertheless, our understanding of whether changes in soil nitrogen could differentially favor some cytotypes over others would benefit from studies done in the field that are also able to examine cytotype responses to a variety of abiotic and biotic environmental conditions (such as temperature, water, CO_2_, herbivore, and pathogen dynamics) that are also changing in concert with changes in soil nitrogen deposition rates.

## CONFLICT OF INTEREST

None declared.

## AUTHOR CONTRIBUTIONS

The experiment was designed and conducted by A. Bales with input from E. Hersch‐Green. Both A. Bales and E. Hersch‐Green collected data, performed data analysis, interpreted the data, and wrote various versions of the manuscript; the final version was approved by both A. Bales and E. Hersch‐Green.

## Data Availability

We intend to make our data accessible online via the Dryad Digital Depository.
