# Supplementary material for: Effects of soil nitrogen on diploid advantage in fireweed, Chamerion angustifolium (Onagraceae)
Source: Ecol Evol. 2018 Dec 26;9(3):1095–109. doi: 10.1002/ece3.4797 (PMC6374662; doi:10.1002/ece3.4797)

Figure S1 – Ploidy and soil nitrogen treatment interaction ($F_{2, 223} = 3.28$, $P = 0.0394$) on (a) aboveground (shoot) and (b) belowground (root) biomass production of fireweed (*Chamerion angustifolium*); full statistical details are reported in Table 2. We report the LS means ± 1 SE's, and significant independent contrasts between cytotypes within nitrogen treatments when significant ($P < 0.05$) with separate letters when a significant interaction between ploidy and nutrient treatment was found.

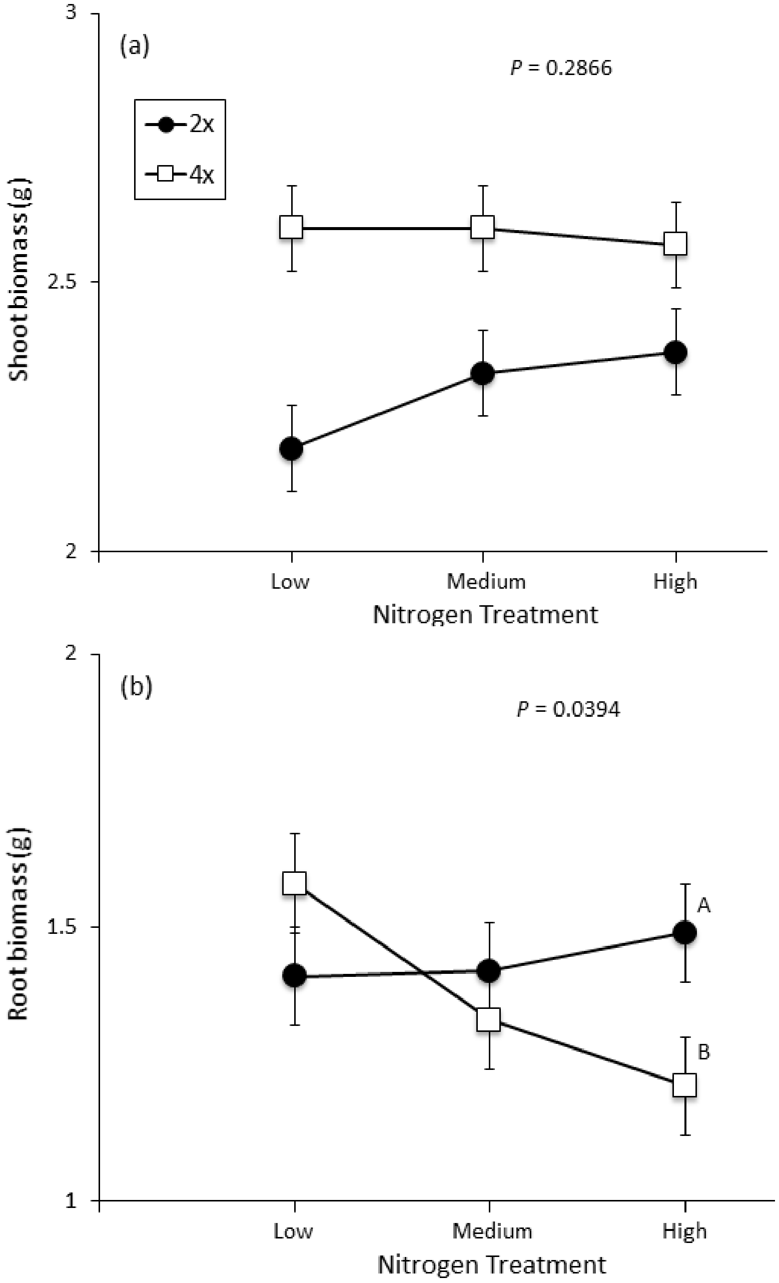

Supplement: Supplementary file 1 [file ECE3-9-1095-s001.pdf]
