# Supplementary material for: Effects of soil nitrogen on diploid advantage in fireweed, Chamerion angustifolium (Onagraceae)
Source: Ecol Evol. 2018 Dec 26;9(3):1095–109. doi: 10.1002/ece3.4797 (PMC6374662; doi:10.1002/ece3.4797)

Figure S2 – Results from fixed effects ANOVAs for the effects of ploidy (diploid, tetraploid), soil N treatment (low, medium, high), their interaction (P x N), and genetic line (nested within ploidy) on the % N in shoots and roots of fireweed (*Chamerion angustifolium*); we report the *F*-ratio and level of significance (Prob > *F*) for the effect of ploidy relative to the between-genetic line variance ($MS_{genetic\ line}$). (a) For shoots: ploidy ($F_{1, 222} = 4.69$, $P = 0.0511$), soil N ($F_{2, 222} = 283.92$, $P < 0.0001$), and genetic line ($F_{12, 222} = 5.19$, $P < 0.0001$) but not the P x N interaction ($F_{2, 222} = 0.41$, $P = 0.6607$) had significant effects on %N. (b) For roots: ploidy ($F_{1, 114} = 8.45$, $P < 0.0001$), soil N ($F_{2, 114} = 200.32$, $P < 0.0001$), the P x N interaction ($F_{2, 114} = 3.83$, $P = 0.0246$), and genetic line ($F_{12, 114} = 2.60$, $P = 0.0042$) all had significant effects on %N in roots. LS means and ±1 SE are reported (error bars).

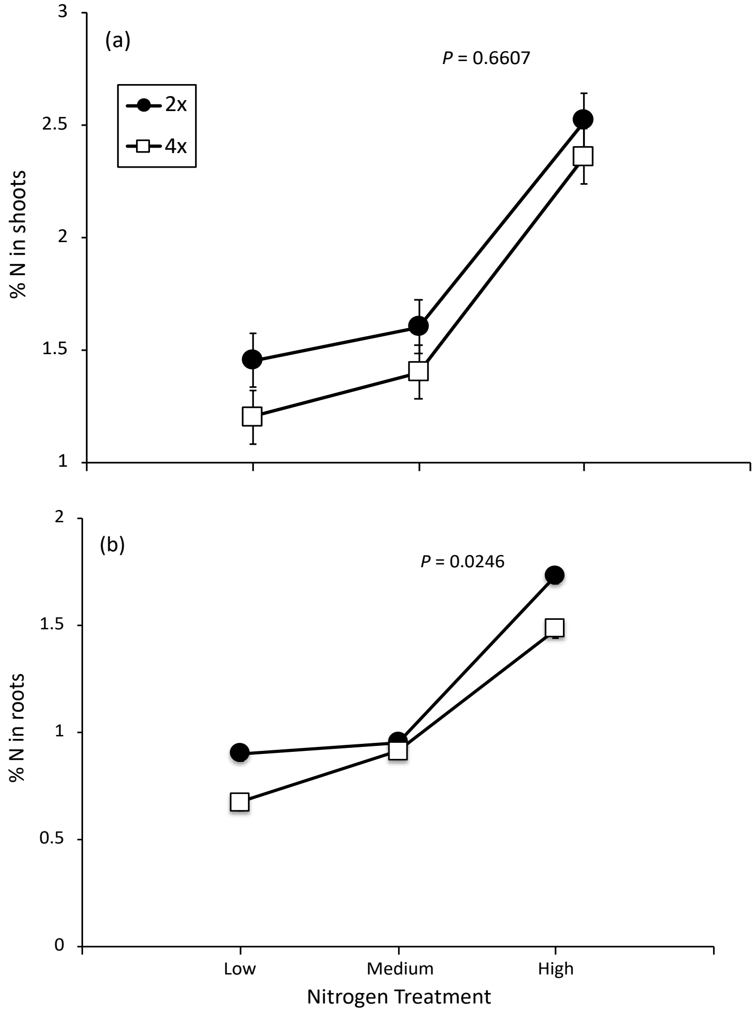

Supplement: Supplementary file 2 [file ECE3-9-1095-s002.pdf]
