# Supplementary material for: Effects of soil nitrogen on diploid advantage in fireweed, Chamerion angustifolium (Onagraceae)
Source: Ecol Evol. 2018 Dec 26;9(3):1095–109. doi: 10.1002/ece3.4797 (PMC6374662; doi:10.1002/ece3.4797)
Supplement: Supplementary file 3 [file ECE3-9-1095-s003.docx]

**Table S1** GPS coordinates of eight locations throughout interior and southern Alaska and the relative number of diploid, triploid, and tetraploid fireweed (*Chamerion angustifolium*) plants identified from each location. * = sites from which we grew diploid and tetraploid seeds for experimentation.

| Location | Latitude | Longitude | Number of plants sampled | Number of cytotypes | | |
| --- | --- | --- | --- | --- | --- | --- |
|  |  |  |  | 2x | 3x | 4x |
| Bonanza Creek * | 64.70369 | -148.29862 | 176 | 33 | 6 | 137 |
| Caribou-Poker Creek * | 65.15275 | -147.48417 | 184 | 139 | 6 | 39 |
| UAF trail system | 64.86248 | -147.86169 | 171 | 23 | 14 | 134 |
| Dog Musher's Park | 64.89780 | -147.72948 | 14 | 0 | 0 | 14 |
| Anchorage/Cook Inlet | 61.19964 | -149.99950 | 69 | 1 | 0 | 68 |
| Turnagain Arm | 60.98097 | -149.42759 | 15 | 0 | 0 | 15 |
| Kenai | 60.64740 | -149.33475 | 28 | 0 | 0 | 28 |
| Seward | 60.09247 | -149.43826 | 27 | 0 | 0 | 27 |
