# Supplementary material for: Effects of soil nitrogen on diploid advantage in fireweed, Chamerion angustifolium (Onagraceae)
Source: Ecol Evol. 2018 Dec 26;9(3):1095–109. doi: 10.1002/ece3.4797 (PMC6374662; doi:10.1002/ece3.4797)
Supplement: Supplementary file 4 [file ECE3-9-1095-s004.docx]

**Appendix S1 –** Fireweed field sampling and flow cytometry methods

We collected leaves and seeds from four fireweed populations in interior Alaska in August 2013 and 2014 and from an additional four populations throughout southern Alaska in August 2014 (Table S1). Leaves and seeds were collected from separate plants, each spaced at a minimum of 15 meters from one another. After collection, leaves were immediately placed into envelopes with desiccant to ensure fast drying of material, whereas fruits were allowed to dry naturally before seeds were collected. We determined ploidy for a total of 684 plants (594 field collected leaf samples and 90 leaf samples from plants grown from different individual field collected seeds).

To determine the ploidy level of field collected seeds, we grew at least two plants from each of 90 different maternal lines (field collected seeds) in the greenhouse at MTU (Dept. of Biological Science, Houghton, MI). We used flow cytometry to estimate plant nuclear 2C DNA content of both dried leaf material collected in the field and live leaf material from the greenhouse to serve as a proxy for cytotype determination. For each plant sampled, we co-chopped a 1-2 cm^2^ piece of fireweed leaf material with a 1-2 cm^2^ piece of a *Solanum lycospersicum* leaf material as an internal standard (*S. lycospersicum* 2C DNA content = 1.96). Leaves were co-chopped in a modified DeLaat’s nuclei isolation buffer with 50 µg ml^-1^ RNase and 50 µg ml^-1^ propidium iodide. Cells were allowed to stain for approximately 40 minutes, and the filtered cell solution was analyzed on an Accuri C6 flow cytometer with CFlow Plus Analysis software (Accuri Inc., Ann Arbor, MI, USA). We used the FL2 detector to measure the relative fluorescence of our samples, and we removed uninformative noise in the data by gating scatter plots to only include particles within the fluorescence range of *C. angustifolium* and *S. lycospersicum* nuclei. We determined ploidy of fireweed using histogram plots showing the relative FL2 fluorescence of *C. angustifolium* and *S. lycospersicum* nuclei counts. We estimated the plant nuclear DNA content by dividing the *C. angustifolium* nuclei fluorescence by the *S. lycospersicum* to obtain a sample:standard ratio. We then multiplied the ratio by the nuclear DNA content of *S. lycospersicum* (1.96 pg) to obtain an estimate of *C. angustifolium* DNA content. The mean nuclear DNA content of diploids, triploids, and tetraploids was 1.48 ± 0.0033 pg, 2.19 ± 0.015 pg, and 2.95 ± 0.0048 pg, respectively. These same methods for flow cytometry were also adopted for plants used in the experiment, although we used fresh leaf material instead of dried leaf material.

In general, we found that most of the sites were dominated by tetraploid cytotypes (seven of eight sites, Table S1) and that triploid cytotypes were rare in all sites. Only the most northern site, Caribou-Poker Creek Basin, was dominated by diploid cytotypes (Table S1). For this experiment, we only used seeds collected from Bonanza Creek and Caribou-Poker Creek Basin (two sites that contrasted in the relative frequencies of diploid and tetraploid cytotypes) and all plants that were found to be triploid were discarded.
